# Improving women's team performance on corners through video training and ball trajectory anticipation

**DOI:** 10.3389/fpsyg.2025.1547493

**Published:** 2025-02-28

**Authors:** Clement Libreau, Nicolas Benguigui

**Affiliations:** ^1^Université de Caen Normandie, Caen, France; ^2^UMR6072 Groupe de Recherche en Informatique, Image, Automatique et Instrumentation de Caen (GREYC), Caen, France

**Keywords:** corner kicks, perceptual training, football, expertise, temporal occultation

## Abstract

The aim of this study was to assess the effectiveness of a video-based perceptual training programme designed to enhance anticipation skills in professional female football players. The video reference task was used to test participants in pre- and post-tests, where they predicted the ball's arrival location during corners. Participants were evenly divided into an experimental group, which received training in the task between the pre- and post-tests using a progressive temporal occlusion method, and a control group, which received no training. The rate of correct responses, response time, and confidence scores were analyzed in the reference task, as well as performance on corners in real matches, to assess the expected transfer of learning to the field. The results revealed that the experimental group, which underwent training, significantly improved their precision in predicting the ball's landing zone after the intervention, with their accuracy score increasing from 54% to 68% (*p* < 0.05, η^2^ = 0.60). Additionally, their response time decreased from 3.2 to 2.4 s (*p* < 0.05, η^2^ = 0.48), and their confidence score improved from 3 to 3.8 (*p* < 0.05, η^2^ = 0.76). This effect slightly diminished after a 6-month retention interval but remained significantly higher than at the pre-test. Furthermore, we observed that the performance of professional football players during corners in actual matches improved, suggesting a positive effect of the video-based perceptual-cognitive training.

## Introduction

Football is characterized by the complexity of its individual and collective interactions. At the individual level, player performance is defined by a multitude of factors, including anthropometric, biomechanical, physiological, psychological, and cognitive aspects. While numerous studies have focused on improving the first set of factors (e.g., Carling et al., 2009; Dellal et al., 2008, 2011; Nédélec et al., 2012), relatively fewer have concentrated on cognitive factors, which encompass perceptual, decision-making, and movement control skills. Applications in this field remain scarce.

A logical starting point for researching the most effective ways to develop cognitive skills is to understand the mechanisms underlying expert advantage. Thanks to research conducted over the past three decades, there is now a substantial database on the perceptual mechanisms that contribute to expertise in sports such as football (Starkes and Allard, 1993). Perceptual-cognitive processes of anticipation and decision-making are key skills linked to performance (Baker et al., 2003). Anticipation is the ability to predict the opponent's action or the ball's movement to gain time in initiating a response (Williams and Ward, 2003). Decision-making is the process of choosing a course of action in a dynamic, uncertain, and complex situation (Causer and Ford, 2014; Silva et al., 2020; Hopwood et al., 2011). These skills are crucial for football performance (Mann et al., 2010; Araújo et al., 2006). During a football match, anticipation and decision-making involve players perceiving and interpreting information related to the ball's position, their teammates, and the opposing players (Travassos et al., 2012; Williams and Ericsson, 2005). Overall, studies on this subject have shown that expert footballers better anticipate their opponents' actions, characterized by more efficient visual search strategies with fewer fixations on more relevant areas, and faster and more accurate responses when faced with occluded video sequences requiring the prediction of the opponent's actions (e.g., Singer et al., 1994; Abernethy et al., 1999). Savelsbergh et al. (2002) conducted a study in which expert and novice goalkeepers had to indicate the arrival zone of penalty kicks presented on a screen by moving a joystick. Expert goalkeepers were generally more accurate in predicting the direction of the penalty kick, waited longer before initiating a response, and made fewer corrective movements with the joystick. They employed a more efficient visual search strategy, characterized by fewer but longer fixations on the most relevant areas, such as the support leg, hips, and ball.

Regarding the ball's trajectory, it is necessary to gather information about it to coordinate one's actions with the ball's arrival and to perform the most appropriate action. However, it does not seem necessary for this information gathering to occur throughout the entire trajectory (Kay, 1957; Whiting and Sharp, 1974; Baurés et al., 2007; Craig et al., 2006). This means that information obtained from an early part of the trajectory can be used to anticipate the ball's path and predict, at least approximately, the location and time of arrival in the interception zone. This is demonstrated in experiments on arrival judgment, where one must predict the moment or location of an object's arrival at a target after it has been occluded (e.g., Benguigui and Bennett, 2010; Makin, 2018; Benguigui et al., 2003).

So far, athlete training aimed at developing anticipation and decision-making skills has been limited to experimental research settings, with very little implementation in field practice. The methods used generally rely on video technology, which has advanced considerably in recent years (e.g., Nelson et al., 2014; Larkin et al., 2016; Hadlow et al., 2018). Players typically respond by verbalizing or writing the most appropriate answer, pressing a button, or moving a joystick (Keller et al., 2018; Van Maarseveen et al., 2018). In summary, these approaches include viewing video sequences of matches or training sessions (Ward and Williams, 2003; Farahani et al., 2020), temporal occlusion (Smeeton et al., 2005; Brenton et al., 2016), providing feedback to participants on the accuracy of their test results (Gorman and Farrow, 2009; Moen et al., 2018; Nelson et al., 2014), and directing attention through video information (Hagemann et al., 2006).

It should be noted that there are two different modes of instruction during perceptual video training, as is the case with training and learning methods in general. The need to identify the best methods for transmitting knowledge has become a central debate in the literature on these topics. Historically, coaches and teachers have extensively used explicit instructions to guide athletes and learners in adapting and progressing. This often meant engaging in learning that relied on highly directive instructions. However, the normative nature of explicit learning has been criticized as unsuitable for developing the flexible and adaptive knowledge systems needed to cope with the uncertainties of the context, particularly in sports (Jackson and Farrow, 2005). Some researchers have proposed that individuals can learn implicitly, without relying on directive instructions about what to consider during learning (e.g., Perruchet and Nicolas, 1998). To promote implicit learning during video-based training sessions, the outcomes of the actions are not shown. The benefits of implicit methods during video-based training for improving anticipation and decision-making have been confirmed in various sports, including football (e.g., Farrow and Abernethy, 2002; Poulter et al., 2005; Roméas et al., 2016). Poulter et al. (2005) examined the effectiveness of explicit and implicit learning paradigms during the early stages of learning the perceptual-motor task of predicting ball direction from temporarily occluded sequences of football penalty kicks. A significant improvement in predicting the ball's horizontal direction (left or right) was observed in the implicit learning group. This group also demonstrated changes in eye movement behavior and an increased awareness of relevant postural cues.

In practical terms, perceptual-cognitive video training relies on the attentional mechanisms that athletes use to focus on early perceptual information sources and on learning the relationship between these characteristics and the relevance of the resulting responses (Faubert and Sidebottom, 2012). A perceptual-cognitive video training programme was developed to improve the perceptual and cognitive performance of Australian football referees in decision-making (Larkin and O'Connor, 2017). After the pre-test, participants were divided into an intervention group and a control group before starting the video training programme, which lasted 12 weeks with one session per week. The videos varied based on the manipulation of the occlusion point. The aim of this manipulation was to gradually increase the difficulty and variety of training sessions to maintain participant engagement. The video training sessions lasted about 20 min and contained between 60 and 225 video clips. The results showed that the decision-making performance of the referees in the intervention group significantly improved. This confirms the value of such video-based perceptual-cognitive training programmes for optimizing decision-making. However, even though it seems highly likely, these results do not necessarily mean that referees will improve their on-field performance. Therefore, the transferability of performance to the field is a key consideration.

Only a few studies have shown perceptual-cognitive training to be beneficial for field performance. Roméas et al. (2016) assessed the transferability of perceptual-cognitive training using a 3D-Multiple-Object-Tracking (MOT) task from a laboratory setting to a football field. During pre- and post-training sessions, they examined three essential skills: passing, dribbling, and shooting, by recording decision-making accuracy. The experimental group underwent 10 sessions of 3D-MOT training, which involved a multi-object tracking task in three dimensions, while the control group received 3D football video training, and a second control group received no specific training. They demonstrated that decision-making accuracy for passing on the field—but not for dribbling and shooting—was higher for the group trained with 3D-MOT compared to the control groups, between the pre- and post-training sessions. This result was correlated with the subjective decision-making accuracy of the players, which was evaluated before and after the sessions using a visual analog scale questionnaire. Additionally, Pagé et al. (2019) analyzed the transferability of performance in decision-making among basketball players. The trained group participated in four training sessions during which they observed basketball video clips presented on a computer screen, while the untrained group received no video training or any other instruction in this area. Decision-making was assessed on the field both before and after the training sessions. In the post-test, the trained group performed significantly better than the control group in terms of speed and decision-making. The results of this study indicate that video-based training can lead to transferable gains in decision-making (see also Gabbett et al., 2008; Rosalie and Müller, 2012; Lorains et al., 2013; Brenton et al., 2019).

Hadlow et al. (2018) hypothesized that the transfer gains potentially obtained from perceptual-cognitive video training could be strongly related to the degree of similarity between the training modality and the real situation. To increase the degree of similarity between the training modality and the real situation, some studies have used video clips that show the participants' point of view (e.g., Ryu et al., 2013; Nimmerichter et al., 2015; Fortes et al., 2021). These videos were filmed from a “first person” perspective, offering a dynamic “self-perception” of the game scene. This approach, when feasible, enhances the fidelity between the video scenes and on-field situations, potentially making them more transferable to performance (Roca and Williams, 2016; Williams, 2002).

Set pieces in football, such as corners, free kicks, penalties, and throw-ins, play a critical role, accounting for 30%−40% of goals scored during a season (Maneiro et al., 2019; Strafford et al., 2019). Among these, corners stand out due to their standardization: the ball is always placed at a fixed position relative to the goal, with a mandatory minimum distance imposed on opposing players. This configuration, combined with their average frequency of 10 per match, makes corners a particularly suitable subject for scientific analysis (Maneiro et al., 2019; Strafford et al., 2019). Studies on performance during corners indicate that they are more decisive in women's football than in men's. For instance, 4.3% of corners result in a goal in women's football, compared to only 2.6% in men's football (De Baranda and Lopez-Riquelme, 2012; Beare and Stone, 2019).

Furthermore, it has been established that specific areas within the penalty box offer higher chances of shooting on goal or scoring. In men's football, these areas include the penalty spot and the far-post zone (e.g., De Baranda and Lopez-Riquelme, 2012; Casal et al., 2015). Conversely, in women's football, the most effective areas are the penalty spot and the near-post zone (Beare and Stone, 2019; Libreau and Benguigui, 2024; Lee and Mills, 2021). These studies highlight the tactical and technical variables that increase the likelihood of success during corners in football. To our knowledge, only one study has attempted to improve a football team's performance during corners by applying the findings of analyses focused on these phases of play. Libreau and Benguigui (2024) developed a field training protocol based on these results. Their findings demonstrated a significant improvement in the team's performance, both in training sessions and in matches. The team trained with this protocol more frequently reached the optimal ball reception zones, attempted shots on goal more often, and scored more goals as a result of the targeted training.

Despite the growing number of studies on video-based perceptual-cognitive training in football, several issues remain, particularly regarding the evaluation of the transfer of performance from controlled laboratory conditions to dynamic field environments. Zhu et al. (2024) emphasize that training programs aimed at improving perceptual-cognitive skills should closely resemble real-world scenarios to promote transfer. However, the majority of studies rely on static or semi-static video presentations, which fail to capture the temporal, spatial, and social complexity of actual matches (Rosalie and Müller, 2012; Lorains et al., 2013). Furthermore, evaluating long-term effects presents a significant challenge. While short-term improvements in anticipation and decision-making are well-documented (Hadlow et al., 2018; Pagé et al., 2019), few studies have explored whether these benefits are maintained months after the intervention. Addressing these issues is crucial for developing more effective and impactful training programs that bridge the gap between laboratory research and on-field performance.

In this context, the objective of the present study was to implement a video training protocol for professional female footballers to improve their performance on corner kicks. This protocol was based on tasks involving the prediction of ball trajectories from “first-person” video footage. We hypothesized that players participating in the perceptual-cognitive video training programme would significantly and durably improve their ability to predict the arrival point of the ball's trajectory and respond more quickly, while also demonstrating greater confidence in their responses. Regarding field application, we hypothesized that this video training protocol would improve the team's performance on corner kicks during matches by increasing their presence in the ball's arrival zone, thereby boosting the percentage of shots on goal and goals scored.

## Method

### Participants

Eighteen professional football players from Montpellier Hérault Sport Club, competing in France's first division for women, voluntarily participated in this study. The participants were divided into two groups (control and experimental). They ranged in age from 19 to 32 years (M = 24.3 years; SD = 3.6). The players involved in offensive corner kicks during the season were assigned to the experimental group, which underwent a pre-test, two post-tests, and predictive training between the pre-test and the first post-test. Those not involved in these corner kicks were placed in the control group, which only took the pre-test and the two post-tests. There was no other specific training on corner kicks for this team during the season. This study, part of a training programme approved by the coaching staff, was also approved by the local ethics committee of the researchers' affiliated university. A consent form was approved and signed by the participants, and the principles of the Declaration of Helsinki were adhered to throughout the study.

### Experimental protocol

The experimental protocol began with a pre-test requiring participants to predict the landing zone of a corner kick after the final part of its trajectory was occluded (see below for a detailed description). This was followed by 12 weeks of video training designed to enhance participants' ability to predict trajectories (see below for more details). This training was exclusive to the experimental group and used the same task as the pre-test. Three days after the final training session, a post-test was conducted, followed by a retention test 6 months later for all participants, under the same conditions as the pre-test. All experimental sessions took place during the season. The perceptual-cognitive training was therefore conducted in addition to the 5–6 weekly practice sessions. The control group did not receive any additional practice.

### Pre-test and post-test phases

**Figure 1:**
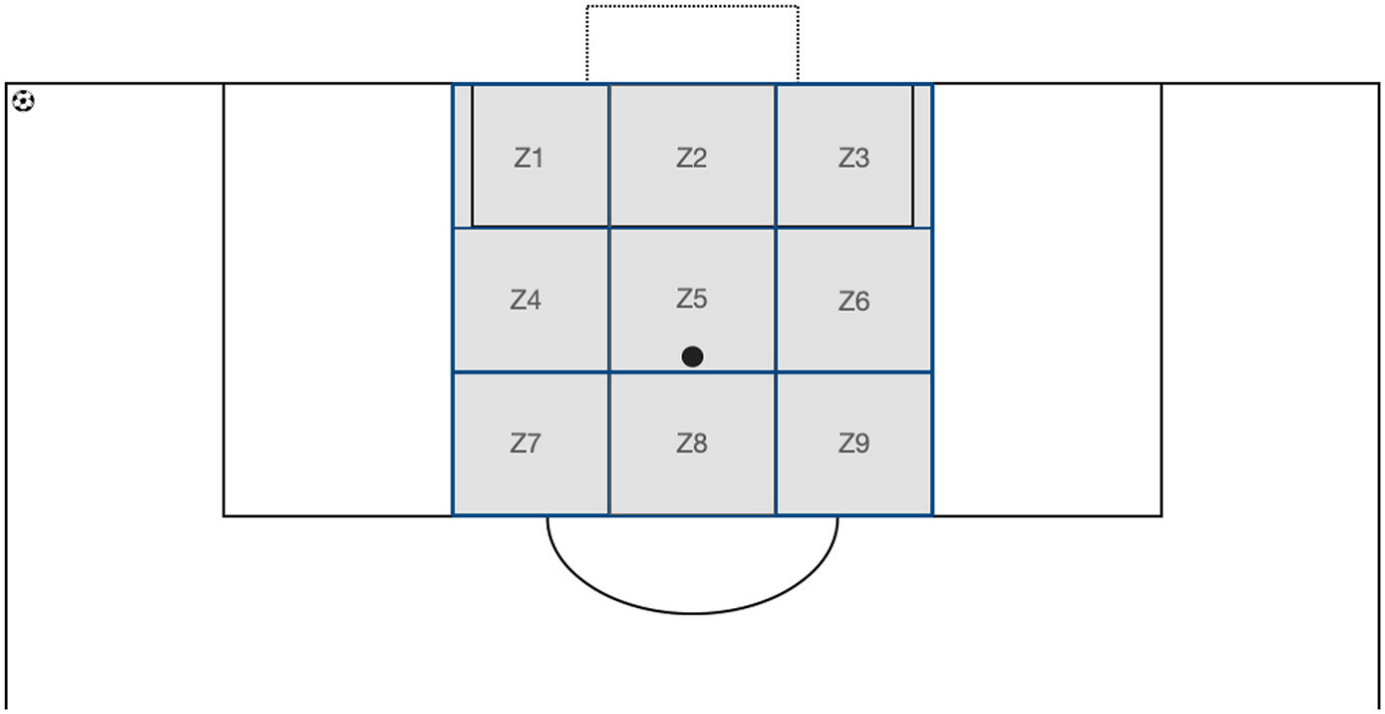
Delivery areas for corners from the left when facing the goal. Each square has sides measuring 5.50 m. For corners arriving from the right, zones Z1, Z4, and Z7 are on the right (at the first post), while zones Z3, Z6, and Z9 are on the left (at the second post). Zones Z2, Z5, and Z8 remain unchanged. For a ball arriving from the right, the zones are reversed, with zone Z1 on the right (at the first post).

The task involved predicting the ball's landing zone among nine possible options after the final part of its trajectory was occluded, by pressing a key on a keyboard (Figure 1). Participants were instructed to respond as accurately and quickly as possible. They were also asked to verbally provide a confidence rating for their response on a scale from 1 to 5. No feedback was given on their responses.

The videos presented were of corner kicks taken by a team member and filmed from the perspective of a receiving player positioned at the entrance and in line with the axis of the penalty area, which is the recommended starting point for arriving in one of the reception zones (Libreau and Benguigui, 2024). The trajectory of the ball could be occluded at 1,200, 800, or 400 ms after the kick to control the amount of information available in each trial. It was not possible to standardize the occlusion time, as the distance of the ball's flight varied from trial to trial depending on the target area. The corners could be taken from either the right or left side of the field, exclusively by right-footed players. A total of 72 different corners were shown to each player: 24 for each presentation time after the kicker's contact with the ball (1,200, 800, and 400 ms), with 8 for each zone. In each occlusion condition, 12 corners were taken from the right side of the field and 12 from the left. For a condition defined by zone, side, and occlusion time, there were one to two trials. Between each corner, a black screen with a 3-s countdown appeared before the kicker's run-up for the next corner.

**Figure 2:**
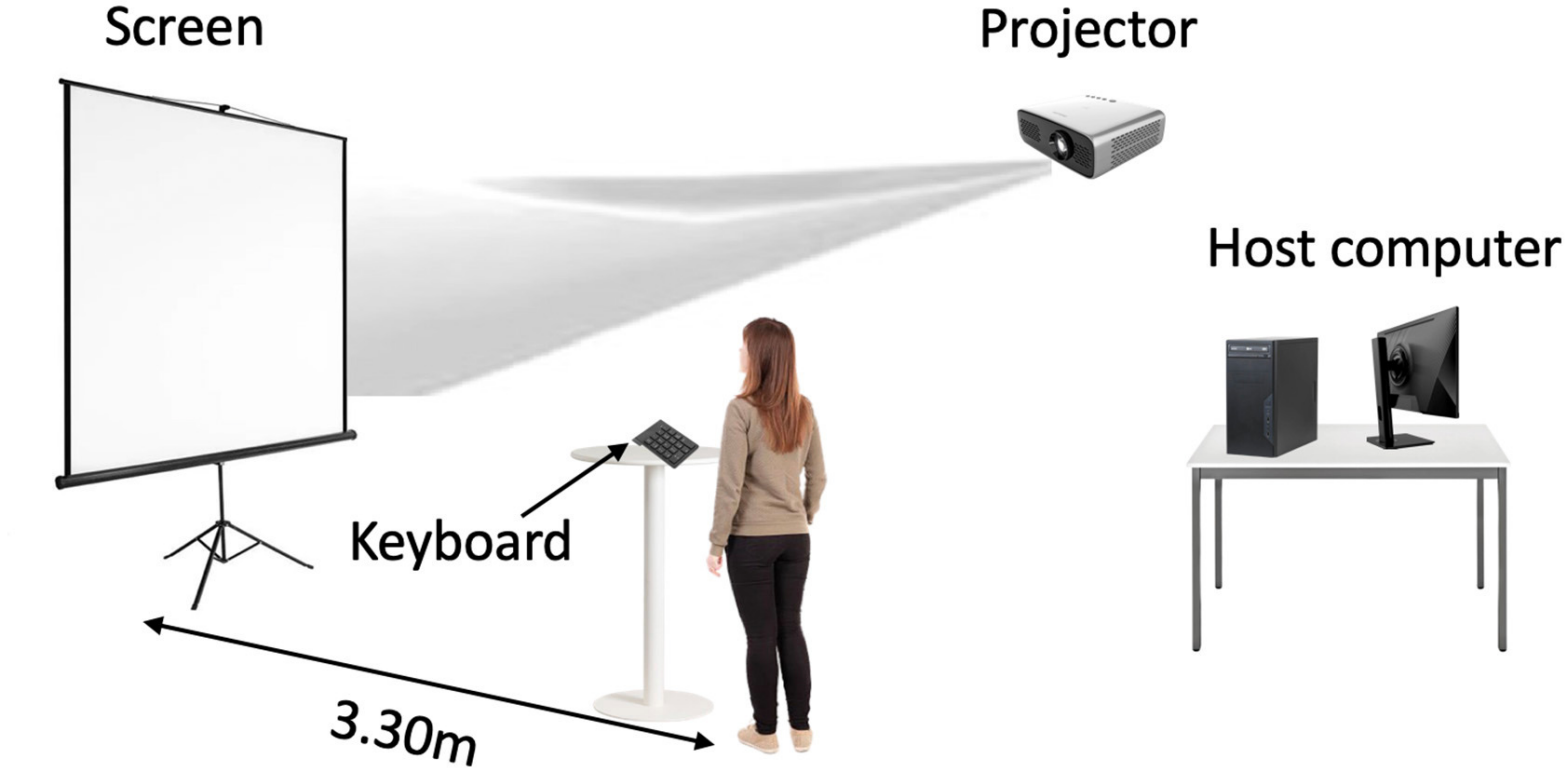
Representation of experimental conditions for tests and training protocol.

Before the pre-test, each participant had a familiarization period of 10 trials to become acquainted with the instructions, equipment, and corner videos (different from those presented during the tests). The sessions took place in a room where the videos were projected onto a wall (3.45 × 2.10 m^2^) using an Epson EB E20 projector. The players stood 3.30 m from the wall to have a viewing angle identical to what they would have on the field. Similarly, the angular size of the kicker and the corner flag matched what they would perceive in a real situation. The player taking the corner kick was thus perceived as on the field, with a 37.5 degree angle. The players had a standing desk in front of them equipped with a Bluetooth wireless numeric keypad featuring nine keys (Satechi brand) and a diagram of the ball landing zones (Figures 1, 2). They were instructed to press the key corresponding to the zone where they believed the ball would land. Key number 1 corresponded to zone 1, key number 2 corresponded to zone 2, and so on.

The videos were displayed, and the responses were recorded using SportsCode^®^ software. The analysis of participants' responses determined whether they accurately identified the ball's landing zone, as well as their response times and confidence scores (rated on a scale of 1–5). The response time was calculated from the moment the ball was occluded (*T* = 0).

### Training phase

The nine players in the experimental group participated in 12 weeks of video training, with one session each week. This amount of practice is consistent with previous studies using video training protocols, which typically include between 5 and 12 sessions (e.g., Fadde, 2006; Mascarenhas et al., 2005; Memmert et al., 2009; Murgia et al., 2014; Schweizer et al., 2011; Farrow and Abernethy, 2002; Gabbett et al., 2008, 2007; Gorman and Farrow, 2009).

The proposed video training protocol was progressive, adjusting the level of difficulty based on the correct response rate achieved in each session. Each training session included 36 trials with corner kick videos filmed under the same conditions as those used in the tests. The video clips used during the training were different from those used during the pre- and post-tests. The database of video clips for this training included a total of 72 different clips, and 36 clips were randomly selected for each training session. Using this procedure, each video clip was presented 6 times over the course of the 12 training sessions, but at different times.

In the first training session, the ball presentation time was 1,400 ms after the corner kick for all videos. If a participant achieved a correct response rate of over 70% regarding the ball's landing zone, she advanced to the next level for the following training session. Each level corresponded to a reduction of 100 ms in ball presentation time.

During the training sessions, participants received feedback on the correct ball landing zone after each response, as is commonly done in video training studies (Abernethy et al., 2012; Catteeuw et al., 2010; Fadde, 2006; Farrow and Abernethy, 2002; Gabbett et al., 2008). No instructions were given to the participants to ensure that they followed an implicit learning protocol (Masters and Maxwell, 2004). Each training session lasted about 15 min.

### Transferability of on-field performance

The video training protocol was conducted during the second half of the 2022/2023 season of the first division of the French women's football championship, from 15 January 2023 to 21 May 2023. To determine whether this video training impacted players' performance in reading the ball's trajectory on offensive corners, we compared the results of corners from the first half of the season (11 September 2022, to 18 December 2022, without video training) to those from the second half (with video training). For this purpose, we created a performance evaluation scale for corners, ranging from 1 to 6, based on the outcome of the corner. A score of 1 corresponded to the absence of offensive players where the ball landed. A score of 2 corresponded to a player being at the ball's landing spot but not touching it because she lost her aerial duel. A score of 3 corresponded to an attacking player gaining possession of the ball but without it heading toward the goal (a pass to a teammate). A score of 4 corresponded to shots directed toward the goal but off-target. A score of 5 corresponded to on-target shots, and a score of 6 corresponded to a goal (Libreau and Benguigui, 2024).

### Data analysis

Data were exported from SportsCode, and descriptive analysis was performed in Microsoft Excel (Version 16.16.27, Microsoft Corporation, United States) to calculate relative frequencies for each variable. The data were further analyzed in SPSS (Version 24.00 SPSS Inc., USA).

Regarding the video training, a Student's *t*-test was used to compare the correct response rates of the two groups for the pre-test, and the results did not reveal any significant effects, indicating that the groups were comparable at the beginning of the experiment. A two-way mixed ANOVA with repeated measures (Groups (Training vs. Control) × Presentation Time (400 vs. 800 vs. 1,200) × Tests (Pre-test vs. Post-test vs. Retention Post-test) was used to analyse the accuracy of ball trajectory predictions, response times, and confidence scores across the different tests. *Post-hoc* Newman-Keuls tests were conducted on significant effects to determine which specific pairs of means differed significantly from each other. The effect size was indicated by eta-squared (η^2^). Based on Cohen's (1992) criteria, the following classifications were adopted: η^2^ < 0.03 = small; 0.03 ≤ η^2^ < 0.10 = moderate; 0.10 ≤ η^2^ < 0.20 = large; and η^2^ ≥ 0.20 = very large effect. The percentages of correct responses were transformed into Fisher's Z-scores for the Student's *t*-tests and ANOVAs. Fisher's z-score is used to extend values close to 100% beyond a fixed boundary and obtain a more normalized distribution.

Regarding the transferability of perceptual-cognitive training to on-field performance in offensive corners, we compared the outcomes of these corners between the two phases of the championship. A Student's *t*-test was used to compare the scores between the two halves of the season, while a Chi-squared test was employed for the percentages in each category. The chi-squared test is a statistical test used to assess whether the observed distribution of a categorical variable differ significantly from what would be expected under the null hypothesis. To assess the reliability and variability of the measurements, we calculated the coefficient of variation [CV = (SD/mean) × 100]. The data were processed using Statistica software, and the alpha level was set at 0.05.

Intra- and inter-observer tests were conducted to assess the reliability of the data collection methods. Intra-observer analysis was conducted by re-evaluating 10% of the corners on two separate occasions, 3 weeks apart, by the principal researcher, a professional football data analyst. Inter-observer analysis followed a similar procedure, involving a second observer who was also a professional football data analyst. The intra- and inter-observer reliability of the data was quantified by calculating Cohen's Kappa (Cohen, 1960). The reliability of each variable is presented with an average kappa statistic of *k* = 0.92 for intra-observer agreement and k = 0.86 for inter-observer agreement, corresponding to “excellent” reliability, respectively (Fleiss et al., 2003).

## Results

### Video training

#### Accuracy of predicting the ball's landing zone based on group, test, and presentation time

**Figure 3:**
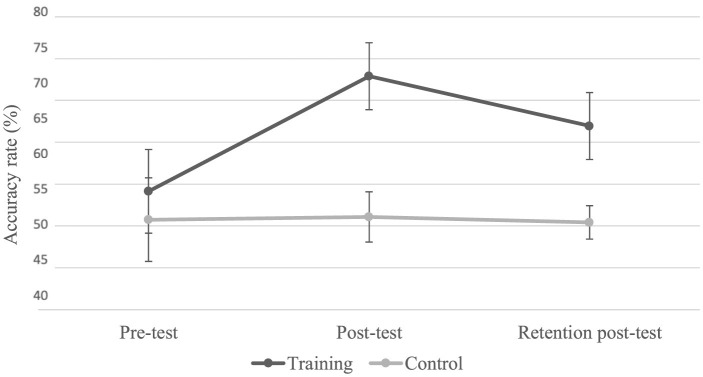
Percentage of correct responses for both groups across tests error bars represent the standard error. The *post-hoc* test indicates a significant improvement in the correct response rate only for the trained group between the pre-test and both post-tests, with no significant difference between the two post-tests.

The analysis of variance revealed significant main effects for Group (*F*(2.24) = 30.57, *p* < 0.05, η^2^ = 0.73), Test (*F*(2.24) = 16.85, *p* < 0.05, η^2^ = 0.6), and Presentation Time (*F*(2.22) = 514.72, *p* < 0.05, η^2^ = 0.97). Additionally, there were significant interactions between Test × Group (*F*(2.22) = 16.56, *p* < 0.05, η^2^ = 0.60; Figure 3 and Table 1) and Test × Presentation Time (*F*(4.44) = 4.43, *p* < 0.05, η^2^ = 0.28; Table 2).

**Table 1:** Results of the significant statistical analyses by group.

	**Groups**	**Pre-test**	**Post-test**	**Retention post-test**
Accuracy in predicting the ball's landing zone (%)	Training	54	68	62
	Control	51	51	50
Response time (s)	Training	3.2	2.4	2.9
	Control	3.3	3.0	3.2
Confidence scores (arbitrary units)	Training	3.0	3.8	3.5
	Control	3.0	3.0	2.8

**Table 2:** Results of the significant statistical analyses by ball trajectory presentation time.

	**Presentation time**	**Pre-test**	**Post-test**	**Retention post-test**
Accuracy in predicting the ball's landing zone (%)	1,200 ms	77	81	78
	800 ms	54	63	61
	400 ms	26	35	28
Response time (s)	1,200 ms	2.8	2.4	2.7
	800 ms	3.0	2.6	3.0
	400 ms	3.9	3.2	3.4
Confidence scores (arbitrary units)	1,200 ms	3.8	4.1	3.9
	800 ms	2.9	3.4	3.1
	400 ms	2.2	2.7	2.4

The Newmann-Keuls *post-hoc* test for the Test × Group interaction indicated a significant improvement in the accuracy rate only for the trained group between the pre-test and both post-tests, with no significant difference between the two post-tests (Figure 3 and Table 1).

The Newmann-Keuls *post-hoc* test for the Test x Presentation Time interaction showed a significant improvement in accuracy between the pre-test and the post-test, but not between the post-test and the retention test for the 1,200 ms condition; a significant improvement between the pre-test and both post-tests for the 800 ms condition; and no difference for the 400 ms condition (Table 2).

### Response time as a function of group, test condition, and presentation time

**Figure 4:**
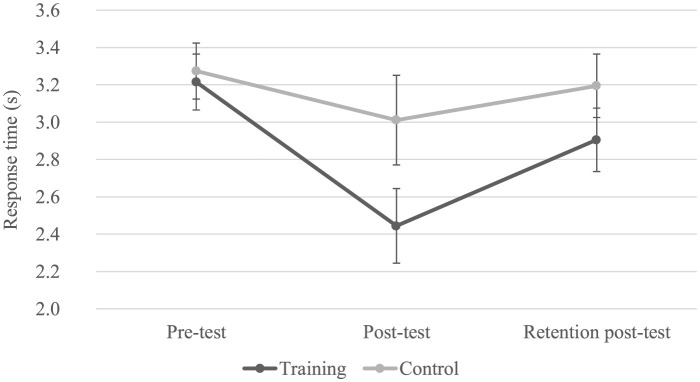
Response time (s) for the two groups by test. Error bars represent the standard error. The *post-hoc* test indicates a significant reduction in response time from the pre-test to both post-tests, but only for the training group.

The analysis of variance reveals a main effect for Group (*F*(1.11) = 38.95, *p* < 0.05, η^2^ = 0.78), Test (*F*(2.22) = 10.15, *p* < 0.05, η^2^ = 0.48), and Presentation Time (*F*(2.22) = 76.01, *p* < 0.05, η^2^ = 0.87). Additionally, interactions were found between Test and Group (*F*(2.22) = 10.15, *p* < 0.05, η^2^ = 0.48; see Figure 4 and Table 1) and between Test and Presentation Time (*F*(4.44) = 3.85, *p* < 0.05, η^2^ = 0.26; Table 2).

The *post-hoc* Newman-Keuls test for the Test × Group interaction shows a significant reduction in response time from the pre-test to both post-tests, but only for the training group (Table 1).

The *post-hoc* Newman-Keuls test for the Test × Presentation Time interaction reveals a significant decrease in response time from the pre-test to the post-test across all three presentation conditions, as well as a significant decrease from the pre-test to the retention post-test for the 400 ms presentation condition. However, no significant reduction in response time was observed between the pre-test and the retention post-test for the 800 and 1,200 ms presentation conditions (Table 2).

### Confidence scores by group, test, and presentation time

**Figure 5:**
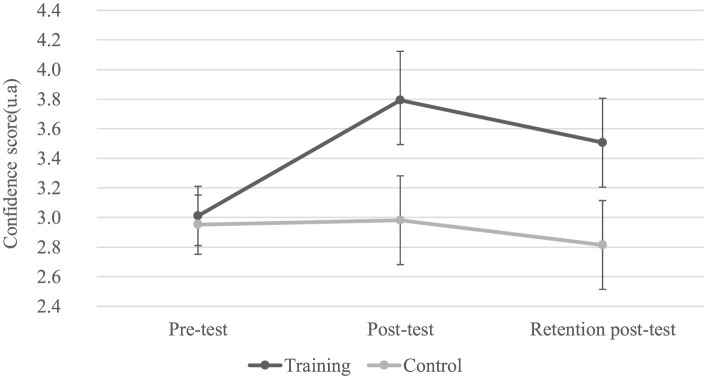
Confidence Scores (arbitrary units) for the two groups by test. Error bars represent the standard error. The *post-hoc* test indicates a significant improvement in confidence scores from the pre-test to both post-tests for the training group, but not for the control group.

The analysis of variance reveals a main effect for the factors Test (*F*(2.22) = 33.98; *p* < 0.05; η^2^ = 0.76), Group (*F*(1.11) = 38.78; *p* < 0.05; η^2^ = 0.78), and Presentation Time (*F*(2.22) = 185.59; *p* < 0.05; η^2^ = 0.94), as well as an interaction between Test and Group (*F*(2.22) = 33.98; *p* < 0.05; η^2^ = 0.76; Figure 5 and Table 1) and an interaction between Test and Presentation Time (*F*(4.44) = 2.57; *p* < 0.05; η^2^ = 0.19; Table 2).

The *post-hoc* Newman-Keuls test for the Test × Group interaction shows a significant improvement in confidence scores from the pre-test to both post-tests for the training group, but not for the control group (Table 1).

The *post-hoc* Newman-Keuls test for the Test × Presentation Time interaction indicates a significant improvement in confidence scores based on presentation time at the post-test, but not at the retention post-test (Table 2).

### Increase in difficulty levels with practice in decreasing presentation time

**Figure 6:**
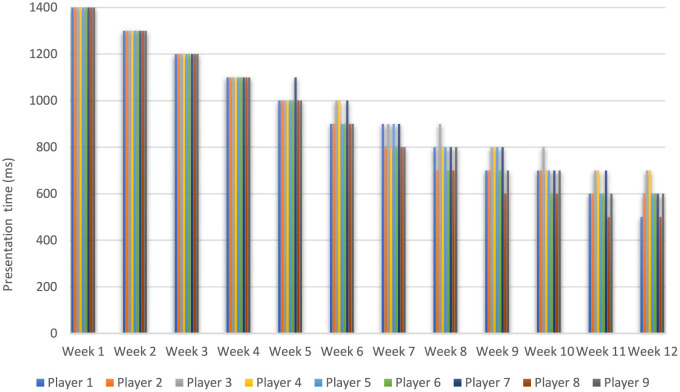
Increase in difficulty levels with practice: evolution of presentation time during training sessions for each player as the presentation time decreases.

Figure 6 illustrates the progression of difficulty levels throughout the training for all players. Similar trends are observed across the board, with some differentiation toward the end of the training, where Players 1 and 8 managed to advance their progress further than the other players.

### Transferability of performance to the field

We also compared the average scores of corner effectiveness between the first and second halves of the season using a Student's *t*-test. The results indicate a significant improvement in performance between these two phases of the championship (*t*(1.28) = 14; *p* < 0.05, η^2^ = 0.48). The average score during the first half was 2.14, compared to 3.00 in the second half.

**Figure 7:**
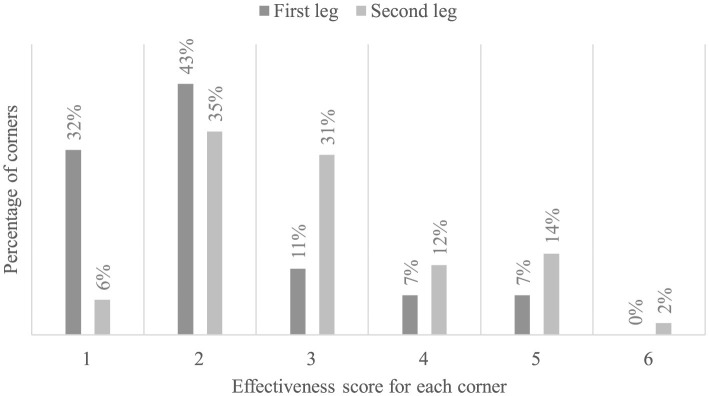
Corner outcome scores between the first and second halves of the season: A score of 1 indicates the absence of offensive players at the ball's landing spot. A score of 2 indicates an offensive player at the landing spot who did not touch the ball due to losing the aerial duel. A score of 3 indicates the ball was recovered by an attacker but did not head toward the goal. A score of 4 indicates missed shots. A score of 5 indicates on-target shots, and a score of 6 indicates a goal.

The results further showed a statistically significant association between the average corner effectiveness scores and the phase of the championship (χ^2^ = 10.6; *p* < 0.05, *V* = 0.245). Corners with a score of 1, meaning no offensive players are near the ball's landing spot, decreased from 32% to 6%. Corners with a score of 2, where an offensive player is present but does not make contact with the ball, decreased from 43% to 35%. Corners where the player makes contact with the ball, but it does not head toward the goal (score of 3), increased from 11% to 31%. Corners with a score of 4, where players take a shot off-target, increased from 7% to 12% between the first and second halves. Corners with a score of 5, where players take an on-target shot, increased from 7% to 14%, and corners with a score of 6, which result in goals, increased from 0% to 2% (Figure 7).

## Discussion

In this study, we tested the effectiveness of video training designed to improve the ability to predict ball trajectories and enhance performance during offensive corners in women's football. This research was conducted with professional female football players, an area that remains relatively underexplored (Beare and Stone, 2019; Lee and Mills, 2021; Libreau and Benguigui, 2024).

Corners in football are complex situations where players need to use the most relevant sources of information to act effectively (Harenberg et al., 2021). As a result, players' perceptual-cognitive skills are highly sought after. It has been suggested that perceptual video training can help improve these skills (e.g., Larkin et al., 2016). In this type of training, participants view interrupted video sequences and must predict the evolution of the situation (e.g., Williams and Grant, 1999; Nimmerichter et al., 2015).

In our study, the test involved a judgment task based on first-person view corner kick videos that were interrupted before the ball reached the target area. The aim was to assess prediction abilities in terms of accuracy and decision-making speed. Participants were also asked to rate their confidence in their responses as a measure of their engagement in potential future match situations. Between the pre-test and post-test, we implemented a 12-week implicit video training protocol. During each training session, participants had to predict the ball's landing area. The protocol included increasing difficulty as players succeeded in the test, achieved by reducing the ball trajectory presentation time.

Our main hypothesis was that this training period would improve prediction abilities for ball trajectories in the video test as well as on the field, leading to better performance on corners in championship matches. The results initially showed high success scores, which were dependent on the presentation time of the trajectory, highlighting the task's relevance and its consistency with previous results showing trajectory prediction abilities (e.g., Benguigui and Bennett, 2010; Makin, 2018; Whiting and Sharp, 1974; Sanderson and Whiting, 1974; Sharp and Whiting, 1974; Whiting et al., 1970).

Results showed an improvement in the percentage of correct responses, response time, and confidence scores in the post-tests exclusively for the trained group. It appears possible to improve prediction accuracy with a video protocol for high-level female football players facing corner trajectories. This finding is consistent with previous studies demonstrating the benefits of video training for perceptual-cognitive mechanisms (Poulter et al., 2005; Gabbett et al., 2008; Núñez et al., 2010; Shafizadeh and Platt, 2012; Ryu et al., 2013; Murgia et al., 2014; Nimmerichter et al., 2015; Fortes et al., 2021). The reduction in response time is particularly notable for a sport like football, which requires quick decision-making. This finding confirms previous research on the topic (Núñez et al., 2009; Nimmerichter et al., 2015). The increase in confidence scores may indicate a potential improvement in decision-making processes.

While the observed performance improvements are promising, it is important to acknowledge the possibility that these gains might be transient. Retention tests are therefore essential to assess whether the benefits of perceptual video training persist over time or diminish after the training period ends. Previous studies have indicated that perceptual video training often leads to short-term skill improvements, which may not necessarily translate into long-term retention (e.g., Núñez et al., 2010; Ryu et al., 2013; Vänttinen et al., 2010). These findings suggest that without reinforcement or carefully designed protocols, the skills developed during training may fade, limiting their practical value in competitive scenarios. A key strength of our study lies in the inclusion of a retention post-test conducted 6 months after the initial post-test. Remarkably, the experimental group maintained significantly higher rates of correct responses, faster response times, and improved confidence scores compared to their baseline and the control group. This sustained improvement suggests a lasting enhancement of anticipation and trajectory prediction abilities, which are critical for high-performance gameplay. The long-term retention observed in our study may be attributed to the specific design of our training protocol. Gradual increases in difficulty, achieved by systematically reducing the ball trajectory presentation time as participants succeeded, likely played a pivotal role in consolidating the learned skills. This aligns with findings by Abernethy et al. (2012) and Savelsbergh et al. (2010), who emphasize the importance of progressively increasing task complexity to promote deeper cognitive engagement and skill automation. By challenging participants with increasingly demanding tasks, our protocol may have facilitated not only the acquisition but also the stabilization of perceptual-cognitive mechanisms.

Another significant innovation of our study is the transferability of the results to match performance over a professional season. Improving ball trajectory prediction abilities in a video protocol does not guarantee that players can apply this skill on the field. To address this, we measured the transfer of training to field performance (Lorains et al., 2013; Larkin et al., 2016; Del Campo et al., 2015). Our study compared corner performance in championship matches before and after implementing video training to identify any potential beneficial effects. Results showed an improvement in average performance scores, particularly in ball receptions and offensive efficiency, as demonstrated in the study by Libreau and Benguigui (2024). Although causality cannot be established due to the multiple factors influencing performance improvement, it is reasonable to assume that our training protocol enhanced anticipation and decision-making abilities for trained players during corners, which partly explains the improved field performance (Nimmerichter et al., 2015; Roméas et al., 2016; Vaeyens et al., 2007). This is supported by the lack of other specific work on this phase of play during the same period. The long duration of the protocol, with increasing difficulty in the video training and the projection of video sequences close to real situations, may explain this improvement in field performance. Another important aspect of our protocol is the use of first-person view videos, which add realism and proximity to real situations (e.g., Poulter et al., 2005; Núñez et al., 2009). It can be assumed that more realistic clips offer significant potential for performance improvement in the field (Roca and Williams, 2016).

After comparing our results with existing literature and highlighting the benefits of perceptual video training, it is important to consider the limitations of this study. First, the participants' response time was over 2 s, which is relatively lengthy compared to the constraints of real game situations, where decisions must be made much faster. This highlights the somewhat artificial nature of the task and the lack of temporal pressure in the video training task. Second, although we aimed to replicate the fidelity of video simulations to real life, some differences remained inevitable, particularly regarding the absence of player movement and scene updates in the training task. The increasing use of virtual reality could address this limitation and enhance task representativeness (Craig, 2013; Fortes et al., 2021).

Secondly, another limitation lies in the composition of the groups: the 9 players in the experimental group were those who regularly participated in offensive corners during official matches, while the 9 players in the control group participated less frequently. This setup could introduce a bias, as the experimental group had greater experience with offensive corners. However, no significant difference was observed between the two groups in the pre-test, which indicates that greater involvement in corners did not provide an initial advantage in predicting the trajectories proposed in our experimental task. On the other hand, we cannot rule out the possibility that this greater involvement in corners led to faster learning during training. The question of the ability to improve performance as a function of the level of expertise is one that could be explored in future experiments.

Lastly, it is not clear whether the improvement in real situations can be directly attributed to video training, and we cannot precisely identify which capabilities players have improved in order to better predict trajectories on the field, in terms of information gathering or visual strategies. This area remains to be explored in order to better understand the mechanisms involved and how to improve training.

In conclusion, our study innovatively demonstrates that video training can enhance the anticipation and decision-making abilities of professional football players during corners, particularly in predicting the ball's arrival zone, with a tangible impact on match performance. These findings represent a significant advancement in the field of perceptual training for ball trajectory analysis and suggest that this approach could complement the physical, technical, and tactical training traditionally implemented by coaches. Given that corners occur ~10 times per match and often play a decisive role (e.g., Strafford et al., 2019), such training programs hold considerable potential for improving team performance. Furthermore, this method could be adapted to other phases of play, such as indirect free kicks or other set-piece situations in team sports.

## Data Availability

The original contributions presented in the study are included in the article/supplementary material, further inquiries can be directed to the corresponding author/s.

## References

[B1] Abernethy B. Schorer J. Jackson R. C. Hagemann N. (2012). Perceptual training methods compared: the relative efficacy of different approaches to enhancing sport-specific anticipation. J. Exp. Psychol. Appl. 18:143. 10.1037/a0028452 22564086

[B2] Abernethy B. Wood J. M. Parks S. (1999). Can the anticipatory skills of experts be learned by novices? Res. Quart. Exerc. Sport 70, 313–318. 10.1080/02701367.1999.10608050 10522289

[B3] Araújo D. Davids K. Hristovski R. (2006). The ecological dynamics of decision making in sport. Psychol. Sport Exerc. 7, 653–676. 10.1016/j.psychsport.2006.07.002

[B4] Baker J. Coté J. Abernethy B. (2003). Learning from the experts: practice activities of expert decision makers in sport. Res. Quart. Exerc. Sport 74, 342–347. 10.1080/02701367.2003.10609101 14510301

[B5] Baurés R. Benguigui N. Amorim M.-A. Siegler I. A. (2007). Intercepting free falling objects: better use Occam's razor than internalize Newton's law. Vision Res. 47, 2982–2991. 10.1016/j.visres.2007.07.024 17884129

[B6] Beare H. Stone J. A. (2019). Analysis of attacking corner kick strategies in the FA women's super league 2017/2018. Int. J. Perform. Anal. Sport 19, 893–903. 10.1080/24748668.2019.1677329

[B7] Benguigui N. Bennett S. J. (2010). Ocular pursuit and the estimation of time-to-contact with accelerating objects in prediction motion are controlled independently based on first-order estimates. Exp. Brain Res. 202, 327–339. 10.1007/s00221-009-2139-0 20039024

[B8] Benguigui N. Ripoll H. Broderick M. P. (2003). Time-to-contact estimation of accelerated stimuli is based on first-order information. J. Exp. Psychol. Hum. Percept. Perform. 29:1083. 10.1037/0096-1523.29.6.1083 14640832

[B9] Brenton J. Müller S. Dempsey A. (2019). Visual-perceptual training with acquisition of the observed motor pattern contributes to greater improvement of visual anticipation. J. Exp. Psychol. Appl. 25:333. 10.1037/xap0000208 30688501

[B10] Brenton J. Müller S. Mansingh A. (2016). Discrimination of visual anticipation in skilled cricket batsmen. J. Appl. Sport Psychol. 28, 483–488. 10.1080/10413200.2016.116222519164835

[B11] Carling C. Le Gall F. Reilly T. Williams A. M. (2009). Do anthropometric and fitness characteristics vary according to birth date distribution in elite youth academy soccer players? Scand. J. Med. Sci. Sports 19, 3–9. 10.1111/j.1600-0838.2008.00867.x 19000100

[B12] Casal C. A. Maneiro R. Ardá T. Losada J. L. Rial A. (2015). Analysis of corner kick success in elite football. Int. J. Perform. Anal. Sport 15, 430–451. 10.1080/24748668.2015.11868805

[B13] Catteeuw P. Gilis B. Jaspers A. Wagemans J. Helsen W. (2010). Training of perceptual-cognitive skills in offside decision making. J. Sport Exerc. Psychol. 32, 845–861. 10.1123/jsep.32.6.845 21282841

[B14] Causer J. Ford P. R. (2014). “Decisions, decisions, decisions”: transfer and specificity of decision-making skill between sports. Cogn. Process. 15, 385–389. 10.1007/s10339-014-0598-0 24414520

[B15] Cohen J. (1960). A coefficient of agreement for nominal scales. Educ. Psychol. Meas. 20, 37–46. 10.1177/001316446002000104

[B16] Cohen J. (1992). Quantitative methods in psychology: a power primer. Psychol. Bull. 112, 155–159. 19565683 10.1037//0033-2909.112.1.155

[B17] Craig C. (2013). Understanding perception and action in sport: how can virtual reality technology help? Sports Technol. 6, 161–169. 10.1080/19346182.2013.855224

[B18] Craig C. M. Berton E. Rao G. Fernandez L. Bootsma R. J. (2006). Judging where a ball will go: the case of curved free kicks in football. Naturwissenschaften 93, 97–101. 10.1007/s00114-005-0071-0 16450083

[B19] De Baranda P. S. Lopez-Riquelme D. (2012). Analysis of corner kicks in relation to match status in the 2006 World Cup. Eur. J. Sport Sci. 12, 121–129. 10.1080/17461391.2010.551418

[B20] Del Campo V. L. Vaíllo R. R. Solana R. S. Hernández F. J. M. (2015). Diferencias en el comportamiento visual y motor de tenistas en laboratorio y en pista de tenis. Rev. Latinoam. Psicol. 47, 136–145. 10.1016/j.rlp.2015.05.003

[B21] Dellal A. Chamari K. Owen A. L. Wong D. P. Lago-Penas C. Hill-Haas S. (2011). Influence of technical instructions on the physiological and physical demands of small-sided soccer games. Eur. J. Sport Sci. 11, 341–346. 10.1080/17461391.2010.521584

[B22] Dellal A. Chamari K. Pintus A. Girard O. Cotte T. Keller D. (2008). Heart rate responses during small-sided games and short intermittent running training in elite soccer players: a comparative study. J. Strength Condit. Res. 22, 1449–1457. 10.1519/JSC.0b013e31817398c6 18714244

[B23] Fadde P. J. (2006). Interactive video training of perceptual decision-making in the sport of baseball. Technol. Instruct. Cognit. Learn. 4, 265–285.

[B24] Farahani J. Soltani P. Rezlescu C. (2020). Assessing decision-making in elite academy footballers using real-world video clips. Prog. Brain Res. 253, 59–70. 10.1016/bs.pbr.2020.06.015 32771130

[B25] Farrow D. Abernethy B. (2002). Can anticipatory skills be learned through implicit video based perceptual training? J. Sports Sci. 20, 471–485. 10.1080/02640410252925143 12137177

[B26] Faubert J. Sidebottom L. (2012). Perceptual-cognitive training of athletes. J. Clin. Sport Psychol. 6, 85–102. 10.1123/jcsp.6.1.85

[B27] Fleiss J. L. Levin B. Paik M. C. (2003). Statistical Methods for Rates and Proportions, 3rd Edn. Hoboken, NJ: John Wiley & Sons Inc.

[B28] Fortes L. S. Almeida S. S. Praça G. M. Nascimento-Júnior J. R. Lima-Junior D. Barbosa B. T.. (2021). Virtual reality promotes greater improvements than video-stimulation screen on perceptual-cognitive skills in young soccer athletes. Hum. Movement Sci. 79:102856. 10.1016/j.humov.2021.102856 34391110

[B29] Gabbett T. Carius J. Mulvey M. (2008). Does improved decision-making ability reduce the physiological demands of game-based activities in field sport athletes? J. Strength Condition. Res. 22, 2027–2035. 10.1519/JSC.0b013e3181887f34 18978606

[B30] Gabbett T. Rubinoff M. Thorburn L. Farrow D. (2007). Testing and training anticipation skills in softball fielders. Int. J. Sports Sci. Coach. 2, 15–24. 10.1260/174795407780367159

[B31] Gorman A. D. Farrow D. (2009). Perceptual training using explicit and implicit instructional techniques: does it benefit skilled performers? Int. J. Sports Sci. Coach. 4, 193–208. 10.1260/174795409788549526

[B32] Hadlow S. M. Panchuk D. Mann D. L. Portus M. R. Abernethy B. (2018). Modified perceptual training in sport: a new classification framework. J. Sci. Med. Sport 21, 950–958. 10.1016/j.jsams.2018.01.011 29433921

[B33] Hagemann N. Strauss B. Cañal-Bruland R. (2006). Training perceptual skill by orienting visual attention. J. Sport Exerc. Psychol. 28, 143–158. 10.1123/jsep.28.2.143

[B34] Harenberg S. Mccarver Z. Worley J. Murr D. Vosloo J. Kakar R. S.. (2021). The effectiveness of 3D multiple object tracking training on decision- making in soccer. Sci. Med. Footb. 6, 355–362. 10.1080/24733938.2021.1965201 35862166

[B35] Hopwood M. J. Mann D. L. Farrow D. Nielsen T. (2011). Does visual-perceptual training augment the fielding performance of skilled cricketers? Int. J. Sports Sci. Coach. 6, 523–535. 10.1260/1747-9541.6.4.523

[B36] Jackson R. C. Farrow D. (2005). Implicit perceptual training: how, when, and why? Hum. Movement Sci. 24, 308–325. 10.1016/j.humov.2005.06.003 16087263

[B37] Kay H. (1957). Information theory in the understanding of skill. Occup. Psychol. 31, 218–224.

[B38] Keller B. S. Raynor A. J. Iredale F. Bruce L. (2018). Tactical skill in Australian youth soccer: does it discriminate age-match skill levels? Int. J. Sports Sci. Coach. 13, 1057–1063. 10.1177/1747954118760778

[B39] Larkin P. O'Connor D. (2017). Talent identification and recruitment in youth soccer: recruiter's perceptions of the key attributes for player recruitment. PLoS ONE 12:e0175716. 10.1371/journal.pone.0175716 28419175 PMC5395184

[B40] Larkin P. O'Connor D. Williams A. M. (2016). Does grit influence sport-specific engagement and perceptual-cognitive expertise in elite youth soccer? J. Appl. Sport Psychol. 28, 129–138. 10.1080/10413200.2015.1085922

[B41] Lee J. Mills S. (2021). Analysis of corner kicks at the FIFA Women's World Cup 2019 in relation to match status and team quality. Int. J. Perform. Anal. Sport 21, 679–699. 10.1080/24748668.2021.1936408

[B42] Libreau C. Benguigui N. (2024). A pioneering study on training attacking corners in women's football. J. Phys. Educ. Sport 24, 1582–1589.

[B43] Lorains M. Ball K. MacMahon C. (2013). Expertise differences in a video decision-making task: speed influences on performance. Psychol. Sport Exerc. 14, 293–297. 10.1016/j.psychsport.2012.11.004

[B44] Makin A. D. J. (2018). The common rate control account of prediction motion. Psychon. Bull. Rev. 25, 1784–1797. 10.3758/s13423-017-1403-8 29197050

[B45] Maneiro R. Casal C. A. Ardá A. Losada J. L. (2019). Application of multivariant decision tree technique in high performance football: the female and male corner kick. PLoS ONE 14:e0212549. 10.1371/journal.pone.0212549 30856199 PMC6411156

[B46] Mann D. L. Abernethy B. Farrow D. (2010). Action specificity increases anticipatory performance and the expert advantage in natural interceptive tasks. Acta Psychol. 135, 17–23. 10.1016/j.actpsy.2010.04.006 20507831

[B47] Mascarenhas D. R. Collins D. Mortimer P. W. Morris B. (2005). Training accurate and coherent decision making in rugby union referees. Sport Psychol. 19, 131–147. 10.1123/tsp.19.2.131

[B48] Masters R. S. W. Maxwell J. P. (2004). The role of explicit and implicit learning in the acquisition of motor skills. Int. J. Sports Sci. Coach. 4, 451–460.

[B49] Memmert D. Simons D. J. Grimme T. (2009). The relationship between visual attention and expertise in sports. Psychol. Sport Exerc. 10, 146–151. 10.1016/j.psychsport.2008.06.002

[B50] Moen F. Hrozanova M. Pensgaard A. M. (2018). The effects of perceptual-cognitive training on subjective performance in elite athletes. Sport J. 21.

[B51] Murgia M. Sors F. Muroni A. F. Santoro I. Prpic V. Galmonte A.. (2014). Using perceptual home-training to improve anticipation skills of soccer goalkeepers. Psychol. Sport Exerc. 15, 642–648. 10.1016/j.psychsport.2014.07.009

[B52] Nédélec M. McCall A. Carling C. Legall F. Berthoin S. Dupont G. (2012). Recovery in soccer: part I—post-match fatigue and time course of recovery. Sports Med. 42, 997–1015. 10.2165/11635270-000000000-00000 23046224

[B53] Nelson L. J. Potrac P. Groom R. (2014). Receiving video-based feedback in elite ice-hockey: a player's perspective. Sport Educ. Soc. 19, 19–40. 10.1080/13573322.2011.613925

[B54] Nimmerichter A. Weber N. J. Wirth K. Haller A. (2015). Effects of video-based visual training on decision-making and reactive agility in adolescent football players. Sports 4:1. 10.3390/sports4010001 29910249 PMC5968940

[B55] Núñez F. J. Oña A. Raya A. Bilbao A. (2009). Differences between expert and novice soccer players when using movement precues to shoot a penalty kick. Perceptual Motor Skills 108, 139–148. 10.2466/pms.108.1.139-148 19425456

[B56] Núñez F. J. Oña A. Raya A. Bilbao A. (2010). Effects of providing advance cues during a soccer penalty kick on the kicker's rate of success. Perceptual Motor Skills 111, 749–760. 10.2466/05.24.25.PMS.111.6.749-760 21319614

[B57] Pagé C. Bernier P. M. Trempe M. (2019). Using video simulations and virtual reality to improve decision-making skills in basketball. J. Sports Sci. 37, 2403–2410. 10.1080/02640414.2019.1638193 31280685

[B58] Perruchet P. Nicolas S. (1998). L'apprentissage implicite: un débat théorique. Psychol. Française 43, 13–26.

[B59] Poulter D. R. Jackson R. C. Wann J. P. Berry D. C. (2005). The effect of learning condition on perceptual anticipation, awareness, and visual search. Human Movement Sci. 24, 345–361. 10.1016/j.humov.2005.06.005 16084616

[B60] Roca A. Williams A. M. (2016). Expertise and the interaction between different perceptual-cognitive skills: Implications for testing and training. Front. Psychol. 7:792. 10.3389/fpsyg.2016.00792 27252677 PMC4879788

[B61] Roméas T. Guldner A. Faubert J. (2016). 3D-Multiple Object Tracking training task improves passing decision-making accuracy in soccer players. Psychol. Sport Exerc. 22, 1–9. 10.1016/j.psychsport.2015.06.002

[B62] Rosalie S. M. Müller S. (2012). A model for the transfer of perceptual-motor skill learning in human behaviors. Res. Quart. Exerc. Sport 83, 413–421. 10.1080/02701367.2012.10599876 22978191

[B63] Ryu D. Kim S. Abernethy B. Mann D. L. (2013). Guiding attention aids the acquisition of anticipatory skill in novice soccer goalkeepers. Res. Quart. Exerc. Sport 84, 252–262. 10.1080/02701367.2013.784843 23930552

[B64] Sanderson F. H. Whiting H. T. A. (1974). Dynamic visual acuity and performance in a catching task. J. Motor Behav. 6, 87–94. 10.1080/00222895.1974.10734984 23952697

[B65] Savelsbergh G. J. Haans S. H. Kooijman M. K. van Kampen P. M. (2010). A method to identify talent: visual search and locomotion behavior in young football players. Hum. Mov. Sci. 29, 764–776. 10.1016/j.humov.2010.05.003 20728954

[B66] Savelsbergh G. J. Williams A. M. Kamp J. V. D. Ward P. (2002). Visual search, anticipation and expertise in soccer goalkeepers. J. Sports Sci. 20, 279–287. 10.1080/026404102317284826 11999482

[B67] Schweizer G. Plessner H. Kahlert D. Brand R. (2011). A video-based training method for improving soccer referees' intuitive decision-making skills. J. Appl. Sport Psychol. 23, 429–442. 10.1080/10413200.2011.555346

[B68] Shafizadeh M. Platt G. K. (2012). Effect of verbal cueing on trajectory anticipation in the penalty kick among novice football goalkeepers. Perceptual Motor Skills 114, 174–184. 10.2466/05.23.25.PMS.114.1.174-184 22582686

[B69] Sharp R. H. Whiting H. T. A. (1974). Exposure and occluded duration effects in a ball-catching skill. J. Motor Behav. 6, 139–147. 10.1080/00222895.1974.10734990 23952726

[B70] Silva A. Gombolay M. Killian T. Jimenez I. Son S. H. (2020). “Optimization methods for interpretable differentiable decision trees applied to reinforcement learning,” in Proceedings of the International Conference on Artificial Intelligence and Statistics (AISTATS 2020), eds. S. Chiappa and R. Calandra [Proceedings of Machine Learning Research (PMLR)].

[B71] Singer R. N. Lidor R. Cauraugh J. H. (1994). Focus of attention during motor skill performance. J. Sports Sci. 12, 335–340. 10.1080/02640419408732179 7932943

[B72] Smeeton N. J. Williams A. M. Hodges N. J. Ward P. (2005). The relative effectiveness of various instructional approaches in developing anticipation skill. J. Exp. Psychol. Appl. 11:98. 10.1037/1076-898X.11.2.98 15998182

[B73] Starkes J. L. Allard F. (eds.). (1993). Cognitive Issues in Motor Expertise. Amsterdam: Elsevier Science.

[B74] Strafford B. W. Smith A. North J. S. Stone J. A. (2019). Comparative analysis of the top six and bottom six teams' corner kick strategies in the 2015/2016 English Premier League. Int. J. Perform. Anal. Sport 19, 904–918. 10.1080/24748668.2019.1677379

[B75] Travassos B. Duarte R. Vilar L. Davids K. Araújo D. (2012). Practice task design in team sports: representativeness enhanced by increasing opportunities for action. J. Sports Sci. 30, 1447–1454. 10.1080/02640414.2012.712716 22871067

[B76] Vaeyens R. Lenoir M. Williams A. M. Philippaerts R. M. (2007). Mechanisms underpinning successful decision making in skilled youth soccer players: an analysis of visual search behaviors. J. Motor Behav. 39, 395–408. 10.3200/JMBR.39.5.395-408 17827116

[B77] Van Maarseveen M. J. Savelsbergh G. J. Oudejans R. R. (2018). In situ examination of decision-making skills and gaze behaviour of basketball players. Hum. Movement Sci. 57, 205–216. 10.1016/j.humov.2017.12.006 29253742

[B78] Vänttinen T. Blomqvist M. Luhtanen P. Häkkinen K. (2010). Effects of age and soccer expertise on general tests of perceptual and motor performance among adolescent soccer players. Perceptual Motor Skills 110, 675–692. 10.2466/pms.110.3.675-692 20681323

[B79] Ward P. Williams A. M. (2003). Perceptual and cognitive skill development in soccer: the multidimensional nature of expert performance. J. Sport Exerc. Psychol. 25, 93–111. 10.1123/jsep.25.1.93

[B80] Whiting H. T. A. Gill E. B. Stephenson J. M. (1970). Critical time intervals for taking in flight information in a ball-catching task. Ergonomics 13, 265−272. 10.1080/00140137008931141

[B81] Whiting H. T. A. Sharp R. H. (1974). Visual occlusion factors in a discrete ball-catching task. J. Motor Behav. 6, 11–16. 10.1080/00222895.1974.10734974 23947405

[B82] Williams A. M. Ericsson K. A. (2005). Perceptual-cognitive expertise in sport: some considerations when applying the expert performance approach. Hum. Movement Sci. 24, 283–307. 10.1016/j.humov.2005.06.002 16095739

[B83] Williams A. M. Grant A. (1999). Training perceptual skill in sport. Int. J. Sport Psychol. 30, 194–220.

[B84] Williams A. M. Ward P. (2003). Perceptual expertise. Expert Perform. Sport 219–249. 10.5040/9781492596257.ch-009

[B85] Williams M. (2002). Perceptual and cognitive expertise in sport. Psychol. 15, 416–417.

[B86] Zhu R. Zheng M. Liu S. Guo J. Cao C. (2024). Effects of perceptual-cognitive training on anticipation and decision-making skills in team sports: a systematic review and meta-analysis. Behav. Sci. 14:919. 10.3390/bs14100919 39457791 PMC11505547

